# Virological failure among people living with HIV receiving second-line antiretroviral therapy in Pune, India

**DOI:** 10.1186/s12879-022-07894-2

**Published:** 2022-12-17

**Authors:** Sonali Salvi, Priyanka Raichur, Dileep Kadam, Shashikala Sangle, Nikhil Gupte, Neetal Nevrekar, Sandesh Patil, Amol Chavan, Smita Nimkar, Ivan Marbaniang, Vidya Mave

**Affiliations:** 1 Department of Medicine, Byramjee Jeejeebhoy Government Medical College and Sassoon General Hospitals, Jai Prakash Narayan Road, Pune, India; 2 Byramjee Jeejeebhoy Government Medical College - Johns Hopkins University Clinical Research Site, BJ Medical College and Sassoon General Hospital, Jai Prakash Narayan Road, Pune, Maharashtra 411001 India; 3 Johns Hopkins University School of Medicine, Baltimore, MD USA; 4 Department of Epidemiology, McGill University, Montreal, QC Canada

**Keywords:** HIV/AIDS, Second-line antiretroviral, Virological failure, Adherence, Protease inhibitors

## Abstract

**Background:**

The number of people receiving second-line antiretroviral therapy (ART) has increased as global access to ART has expanded. Data on the burden and factors associated with second-line ART virologic failure (VF) from India remain limited.

**Methods:**

We conducted cross-sectional viral load (VL) testing among adults (≥ 18 years) who were registered at a publicly funded ART center in western India between 2014 and 2015 and had received second-line ART for at least 6 months. Sociodemographic and clinical characteristics were abstracted from routinely collected programmatic data. Logistic regression evaluated factors associated with VF (defined as VL > 1000 copies/mL).

**Results:**

Among 400 participants, median age was 40 years (IQR 34–44), 71% (285/400) were male, and 15% (59/400) had VF. Relative to participants without VF, those with VF had lower median CD4 counts (230 vs 406 cells/mm^3^, p < 0.0001), lower weight at first-line failure (49 vs 52 kg, p = 0.003), were more likely to have an opportunistic infection (17% vs 3%, p < 0.0001) and less likely to have optimal ART adherence (71% vs 87%, p = 0.005). In multivariable analysis, VF was associated with opportunistic infection (aOR, 4.84; 95% CI, 1.77–13.24), lower CD4 count (aOR 4.15; 95% CI, 1.98–8.71) and lower weight at first-line failure (aOR, 2.67; 95% CI, 1.33–5.34).

**Conclusions:**

We found second-line VF in about a sixth of participants in our setting, which was associated with nearly fivefold increased odds in the context of opportunistic infection. Weight could be a useful clinical indicator for second-line VF.

**Supplementary Information:**

The online version contains supplementary material available at 10.1186/s12879-022-07894-2.

## Introduction

Globally, the prevalence of failure of first-line antiretroviral therapy (ART) regimens for adult HIV infection ranges between 20% and 82% [1]. This has concurrently increased the need for countries to expand the rollout of second-line ART regimens. In India, approximately 50,000 people living with HIV (PLHIV) are on second-line ART [2]. However, country-specific estimates of second-line ART failure and associated factors have not been well described.

The National AIDS Control Organization (NACO), India’s premier HIV program has provided publicly funded second-line ART since 2008 [3]. Second-line ART regimens in India have evolved with recommendations of the World Health Organization’s (WHO’s) guidelines. Prior to 2019, Protease Inhibitor (PI)-based regimens were the mainstay of second-line ART, comprising a ritonavir (r)-boosted PI atazanavir (ATV/r) or lopinavir (LPV/r); with ATV/r as the PI of choice, and two nucleoside reverse transcriptase inhibitors (NRTI) [Tenofovir disoproxil fumarate (TDF)/abacavir [ABC] + Lamivudine (3TC)] [4]. Since 2019, regimens comprising the integrase strand transfer inhibitor (INSTI) dolutegravir (DTG) have been introduced (together with two NRTI: zidovudine (AZT)/TDF + 3TC) [5].

Prior to 2018, viral load (VL) monitoring in India was targeted only at PLHIV that were suspected to have immunological failure on first-line ART [4]. Since 2018, NACO has designated VL as the preferred approach to monitor treatment response to second-line ART, with testing recommended at 6 months, 12 months, and yearly thereafter, following second-line ART initiation [6, 7]. The prevalence of virological failure (VF, i.e., high VL) on second-line ART reported across 19 studies from low-and-middle income countries (LMICs) included in a meta-analysis, was between 6% and 38%. This meta-analysis of 2035 PLHIV found poor adherence to second-line ART to be the primary factor associated with VF [8]. Older age, lower CD4 counts, and socioeconomic disadvantage are other factors reported to be associated with second-line VF [9–11]. A limitation of these studies is that none of them included data from India, making the generalizability of their findings to the country questionable.

Third-line ART regimens (following second-line failure) constitute the last line of treatment for HIV. To identify geographically relevant strategies to reduce the number of people that will require third-line ART, it is important to generate data on second-line ART failure. Here, we evaluate the prevalence of, and factors associated with VF among PLHIV receiving second-line ART under programmatic conditions in western India.

## Methods

### Study design, study site and study population

This was a convenience sample of PLHIV aged ≥ 18 years registered for HIV care at the ART center affiliated with Byramjee Jeejeebhoy Government Medical College and Sassoon General Hospitals (BJGMC-SGH).

The BJGMC-SGH ART center was established in 2004 in Pune, Maharashtra (a state in western India). Maharashtra has one of the highest HIV burdens in India, with approximately 330,000 PLHIV, and the adult HIV prevalence is higher than the national average (0.36% vs 0.22%) [12]. The ART center currently has over 5000 PLHIV in active care, including approximately 1800 receiving second-line ART.

Eligible PLHIV were those that had received second-line ART for ≥ 6 months between August 2014 and September 2015 due to first-line ART failure [4]. PLHIV receiving second-line regimens as an alternate regimen due to toxicity of the first-line regime were excluded. We conducted cross-sectional VL testing among all consenting eligible PLHIV, henceforth called study participants. Since study accrual occurred prior to 2019, all study participants in this analysis exclusively received PI-based (ATV/r or LPV/r) second line ART.

### Data variables and definition

Details of all PLHIV registered for care under India’s national HIV care and treatment program are routinely collected using a standard paper form and subsequently entered into an electronic database. VL tests conducted through the study were linked to this database.

#### Outcome

Consistent with published literature, we defined VF as VL > 1000 copies/mL at a single time point [13, 14].

#### Predictors

These were variables hypothesized to be associated with the outcome, and available to be abstracted from the electronic database. Predictors included (a) sociodemographic characteristics: age, sex, income, education, occupation, marital status, history of smoking and alcohol consumption and (b) clinical characteristics: time-updated CD4 count, years on first and second-line ART, past and present opportunistic infections (OIs), time-updated WHO HIV stage, weight at first-line failure, ART regimen and adherence.

Median weight was used to dichotomize weight at first-line failure into two categories (< 51 kg, ≥ 51 kg). Past OI were those occurring before second-line ART initiation, and present OI were those occurring after second-line ART initiation. Treatment adherence was calculated as percentage of pills consumed to those prescribed. Adherence was categorized as suboptimal (< 95%) or optimal (> 95%), consistent with NACO and WHO guidelines [7, 15].

### Statistical analysis

We used Wilcoxon rank sum test and Fisher’s exact test to compare continuous and categorical variables, respectively. Statistical significance was set to a two-sided p-value of 0.05. Associations between VF and the predictors were assessed using univariate and multivariable logistic regression. Multivariable models were adjusted for age, sex and variables found to be significant in univariate analysis.

All statistical analyses were performed with STATA^®^ version 14.2 (STATA Corp, College Station, TX, USA).

### Ethics approval and consent to participate

The Institutional Review Board of Johns Hopkins University and the Ethics Committee of BJGMC-SGH approved the study (Approval number IRB00026444). Written informed consent for VL testing was obtained from all participants.

## Results

### Study population and characteristics

Among 400 PLHIV enrolled, median age was 40 years [interquartile range (IQR) 34–44], 71% (285/400) were male, median time-updated CD4 count was 385 cells/mm^3^ (IQR 228–547) and median VL was 42.5 copies/mL (IQR 40–299). Second-line ART was initiated at a median 4 years (IQR 3–6) after first-line ART initiation. A majority (96%, 385 of 400) were on ATV/r-based regimen. Past OI was reported by 57% (224/400) and 5% (20/400) reported a present OI. Tuberculosis (TB) was the most reported past (135/224) and present (8/20) OI (Additional file 1: Table S1). Suboptimal adherence was observed in 15% (62/400) of study participants (Table 1).

**Table 1:** Characteristics of adult PLHIV on second-line ART at BJGMC-SGH ART center, August 2014–September 2015

Characteristics	Overall (n = 400)	Virological failure^a^ (n = 59)	No virological failure (n = 341)	*p*
Age				
Median, y (IQR)	40 (34–44)	38 (33–42)	40 (35–45)	0.10
< 40	189 (47.3)	30 (50.8)	159 (46.6)	0.57
≥ 40	211 (52.7)	29 (49.2)	182 (53.4)	
Sex				
Male	285 (71.3)	42 (71.2)	243 (71.3)	> 0.95
Female	115 (28.7)	17 (27.8)	98 (28.7)	
Time-updated CD4 count, cells/mm^3^				
Median (IQR)	385 (228–547)	230 (114–354)	406 (251–562)	< 0.0001
> 350	223 (55.7)	16 (27.1)	207 (60.7)	< 0.0001
200–350	95 (23.8)	21 (35.6)	74 (21.7)	
< 200	82 (20.5)	22 (37.3)	60 (17.6)	
Median time on ART, y (IQR)				
Overall	7 (6–9)	7 (5–9)	7 (6–9)	0.30
First-line ART	4 (3–6)	4 (3–6)	4 (3–6)	0.36
Second line ART	3 (2–3)	3 (2–3)	3 (2–3)	0.73
< 3 years	165 (41.2)	25 (42.4)	140 (41.1)	0.89
≥ 3 years	235 (58.8)	34 (57.6)	201 (58.9)	
Marital status				
Not married	83 (20.9)	12 (20.3)	71 (21.0)	> 0.95
Married	314 (79.1)	47 (79.7)	267 (79)	
Education level				
Illiterate and primary	105 (26.4)	18 (31.6)	87 (25.6)	
Secondary	219 (55.2)	30 (52.6)	189 (55.6)	0.62
College and above	73 (18.4)	9 (15.8)	64 (18.8)	
Income category, Indian rupees^b^				
< 5000	225 (61.0)	34 (57.6)	191 (61.6)	0.41
5000–10,000	120 (32.5)	23 (39.0)	97 (31.)	
> 10,000	24 (6.5)	2 (3.4)	22 (7.1)	
Employed				
No	88 (22.4)	14 (23.7)	74 (22.6)	0.87
Yes	305 (77.6)	45 (76.3)	260 (77.8)	
Smoking				
No	323 (83.9)	53 (89.8)	270 (82.8)	0.25
Yes	62 (16.1)	6 (10.2)	56 (17.2)	
Alcohol use				
No	311 (80.8)	50 (86.2)	261 (79.8)	0.28
Yes	74 (19.2)	8 (13.8)	66 (18.2)	
Past opportunistic infection^c^				
No	170 (43.2)	26 (44.1)	144 (43.9)	0.88
Yes	224 (56.8)	33 (55.9)	191 (56.1)	
Present opportunistic infection^d^				
No	379 (95.0)	49 (83.1)	330 (97.1)	< 0.0001
Yes	20 (5.0)	10 (16.9)	10 (2.9)	
WHO stage at first-line failure				
I	342 (85.5)	46 (78.0)	296 (86.8)	0.24
II	20 (5.0)	5 (8.5)	15 (4.4)	
III	22 (5.5)	5 (8.5)	17 (4.9)	
IV	16 (4.0)	3 (5.1)	13 (3.8)	
Weight at first-line failure, kg				
Median (IQR)	51 (45–59)	49 (42–54)	52 (45–60)	0.003
< 51	193 (48.3)	39 (66.1)	154 (45.2)	0.003
≥ 51	207 (51.7)	20 (33.9)	187 (54.8)	
Adherence > 95%^e^				
No	62(15.5)	17 (28.8)	45 (13.2)	0.005
Yes	338 (84.5)	42 (71.2)	296 (86.8)	
Protease inhibitor				
Lopinavir/ritonavir	15 (3.7)	1 (2.0)	14 (4.1)	0.70
Atazanavir/ritonavir	385 (96.3)	58 (98.0)	327 (95.9)	
ART regimen at first-line failure				
AZT/3TC/NVP	209 (52.2)	26 (44.1)	183 (53.7)	0.32
d4T/3TC/NVP	98 (24.5)	13 (22.03)	85 (24.9)	
AZT/3TC/EFV	29 (7.3)	6 (10.1)	23 (6.7)	
d4T /3TC/EFV	19 (4.7)	5 (8.5)	14 (4.1)	
TDF/3TC/NVP	17 (4.2)	3 (5.1)	14 (4.1)	
TDF/3TC/EFV	10 (2.5)	1 (1.7)	9 (2.6)	
Unknown	18 (4.6)	5 (8.5)	13 (3.9)	

Data are presented as n (%), unless otherwise indicated

*PLHIV* people living with HIV, *ART* antiretroviral therapy, *BJGMC-SGH* Byramjee Jeejeebhoy Government Medical College and Sassoon General Hospitals, *IQR* interquartile range, *WHO* World Health Organization, *AZT* Zidovudine, *3TC* Lamivudine, *NVP* Nevirapine, *d4T* Stavudine, *EFV* Efavirenz, *TDF* Tenofovir disoproxil fumarate

^a^Defined as viral load > 1000 copies/mL after at least 6 months on a second-line regimen

^b^5000–1000 INR =  ~ 64–130 USD (As per May 2022 exchange rate)

^c^Defined as occurrence of opportunistic infection (including primarily Pulmonary Tuberculosis, Diarrhoea, Herpes Zoster, Candidiasis, Weight loss, Cryptococcal Meningitis, Others) prior to initiation of second line ART

^d^Defined as occurrence of opportunistic infection after initiation of second line ART

^e^Calculated as the number of pills consumed divided by the number of pills prescribed multiplied by 100

### Prevalence of virological failure

VF was observed in 15% (59/400) of study participants. Compared to participants without VF, those with VF had a lower median CD4 count [230 cells/mm^3^ (IQR 114–354) vs 406 cells/mm^3^ (IQR 251–562), p < 0.0001], lower median weight at first-line failure [49 kg (IQR 42–54) vs 52 kg (IQR 45–60), p = 0.003], were more likely to have present OI (17% vs 3%, p < 0.0001) and suboptimal adherence (29% vs 13%, p = 0.005) (Table 1). Half of the participants with VF and present OI (n = 05/10) had suboptimal adherence.

### Associations of virological failure with predictors

In univariate analysis VF was associated with: (a) time-updated CD4 count level ≤ 350 cells/mm^3^ [Odds ratio [OR] for 200–350 cells/mm^3^: 3.67; 95% confidence interval (CI) 1.82–7.41 and OR for < 200 cells/mm^3^: 4.74; 95% CI, 2.34–9.60]; (b) present OI (OR 6.73; 95% CI 2.66–17); (c) weight < 51 kg at first-line failure (OR 2.36; 95% CI 1.32–4.22); and (d) suboptimal adherence (OR 2.66; 95% CI 1.39–5.07) (Table 2).

**Table 2:** Risk factors for virologic failure on second-line ART in univariate and multivariable logistic regression models

Characteristic	Univariate analysis		Multivariable analysis^a^	
	OR (95% CI)	*p*	aOR (95% CI)	*p*
Age	0.97 (0.94–1.003)	0.08		
< 40	Ref.		Ref.	
≥ 40	0.84 (0.49–1.47)	0.55	0.77 (0.42–1.41)	0.40
Gender				
Male	Ref.		Ref.	
Female	1.01 (0.55–1.85)	> 0.95	0.62 (0.29–1.30)	0.21
Time-updated CD4 count, cells/mm^3^ (per 50 cells/mm^3^ increase)	0.83 (0.77–0.90)	< 0.0001		
Time-updated CD4 count, cells/mm^3^				
> 350	Ref.		Ref.	
200–350	3.67 (1.82–7.41)	< 0.0001	3.39 (1.63–7.05)	0.001
< 200	4.74 (2.34–9.60)	< 0.0001	4.15 (1.98–8.71)	< 0.0001
Time on second-line ART, y				
≥ 3	Ref.			
< 3	1.05 (0.59–1.85)	0.85		
Marital status				
Not married	Ref.			
Married	1.04 (0.53–2.08)	0.91		
Education level				
Illiterate and primary	Ref.			
Secondary	0.77 (0.41–1.45)	0.42		
College and above	0.67 (0.29–1.61)	0.38		
Income, Indian rupees^b^				
< 5000	Ref.			
5000–10,000	1.33 (0.74–2.39)	0.34		
> 10,000	0.51 (0.11–2.27)	0.38		
Employed				
No	Ref.			
Yes	0.91 (0.48–1.75)	0.79		
Smoking				
No	Ref.			
Yes	0.55 (0.23–1.34)	0.18		
Alcohol use				
No	Ref.			
Yes	0.63 (0.29–1.40)	0.26		
Past opportunistic infection^c^				
No	Ref.			
Yes	0.96 (0.55–1.67)	0.88		
Present opportunistic infection^d^				
No	Ref.		Ref.	
Yes	6.73 (2.66–17.00)	< 0.0001	4.84 (1.77–13.24)	0.002
WHO stage at first-line failure				
I	Ref.			
II	2.14 (0.74–6.18)	0.16		
III	1.90 (0.67–5.38)	0.23		
IV	1.48 (0.41–5.41)	0.55		
Weight at first line failure	0.95 (0.92–0.98)	0.004		
Weight at first-line failure, kg				
≥ 51	Ref.		Ref.	
< 51	2.36 (1.32–4.22)	0.004	2.67 (1.33–5.34)	0.005
Adherence > 95%^e^				
Yes	Ref.		Ref.	
No	2.66 (1.39—5.07)	0.003	1.79 (0.86–3.67)	0.12
Protease inhibitor				
Lopinavir/ritonavir	Ref.			
Atazanavir/ritonavir	2.48 (0.32–19.2)	0.38		

*ART* antiretroviral therapy, *aOR* adjusted odds ratio, *IQR* interquartile range, *OR* odds ratio, *WHO* World Health Organization

^a^Adjusted for age, sex, time-updated CD4 count, present opportunistic infection, weight at first-line failure, and adherence > 95%

^b^5000–1000 INR =  ~ 64–130 USD (As per May 2022 exchange rate)

^c^Defined as occurrence of opportunistic infection (including primarily Pulmonary Tuberculosis, Diarrhoea, Herpes Zoster, Candidiasis, Weight loss, Cryptococcal Meningitis, Others) prior to initiation of second line ART

^d^Defined as occurrence of opportunistic infection after initiation of second line ART

^e^Calculated as the number of pills consumed divided by the number of pills prescribed multiplied by 100

In multivariable analysis adjusted for age, sex, time-updated CD4 count, present OI, weight at first-line failure and adherence, (a) CD4 count level ≤ 350 cells/mm^3^ [adjusted OR (aOR), for 200–350 cells/mm^3^: 3.39; 95% CI, 1.63–7.05), and aOR for CD4 count < 200 cells/mm^3^: 4.15; 95% CI, 1.98–8.71], (b) present OI (aOR 4.84; 95% CI 1.77–13.24) and (c) weight < 51 kg at first-line failure (aOR 2.67; 95% CI 1.33–5.34) remained significantly associated with VF (Table 2).

## Discussion

Our cross-sectional study found that around one-sixth of participants met criteria for VF on second-line ART at a large tertiary ART center in western India. In addition, our analysis identified significant associations between VF and lower time-updated CD4 count (≤ 350 cells/mm^3^), present OI and low weight (< 51 kg). Overall, this study adds to the limited evidence on second-line VF and associated factors in India.

The prevalence of VF in our study population is consistent with that reported in the only other study from north India on second-line ART associated VF [3]. However, our estimate was obtained using a larger sample size from a different geographical region in India with higher HIV prevalence. Additionally, compared to the study from north India that included data only till 2012, the data we use is updated to 2015. Our prevalence estimate is also consistent with reports of VF from other LMICs, although varying definitions of VF across studies, makes it challenging to directly compare the burden of second-line associated VF across countries [9].

Similar to previous studies, our analysis indicates that low CD4 count at the time of VL testing is associated with VF [9, 10]. The cross-sectional nature of our data precludes us from commenting on the directionality of this association. The WHO has recommended that in situations where VL measurement is routinely available and PLHIV are clinically stable on ART, VL be exclusively used to monitor second-line ART treatment outcomes [16]. However, in many LMICs, the regularity of VL testing remains suboptimal. A recent study from Asia of 31,346 PLHIV (with > 60% of the data originating from India) showed that VL testing was performed less than annually in > 50% of PLHIV [17]. At present, NACO recommends CD4 monitoring among adult PLHIV on second-line ART with CD4 count < 350 cells/mm^3^ in addition to 6-monthly VL testing [6, 7]. Given the implementational challenges of regular VL testing in India, CD4 count measurement will continue to remain an important aspect of monitoring PLHIV on second-line ART and may be useful to identify PLHIV at potential risk of VF.

The presence of an OI at the time of diagnosis of VF was associated with second-line failure in our cohort. However, as this was a cross-sectional study, it was not possible to assess whether OIs were the cause or effect of VF. As in previous studies from India, TB was the most common OI [18, 19]. As per NACO guidelines, PLHIV diagnosed with TB are treated with rifabutin-based antituberculosis therapy, and the additional pill burden could affect adherence in this subset of PLHIV [20]. Our analysis indicates suboptimal adherence to ART among half of PLHIV with second-line VF and an OI. These findings suggest that patients with active TB on second-line ART may require additional adherence support.

We found that lower weight at first-line failure was associated with second-line VF. A study from Ethiopia reported that weight loss after starting second-line ART was associated with treatment failure [21]. The measurement of weight is easy and inexpensive to perform. Therefore, in LMICs, where the burden of HIV is high and resources are limited, it may be of value for future studies to better understand the role of weight as a predictor of treatment response to second-line ART.

There are several limitations to our study findings. A major limitation is that we used a data set that does not include DTG-based second-line therapy. Currently 80% of PLHIV at BJGMC-SGH ART center receiving second-line ART are on PI-based regimens, making these findings relevant to a majority. Moreover, as recently as September 2022, there were DTG stockouts across India increasing the reliance on PI-based second-line regimens [22]. As already mentioned, to our knowledge, this remains the most updated dataset from India to describe VF among those receiving second-line ART. Future analyses should work on updating our findings. We used a single VL measurement to define VF. WHO defines second-line treatment failure as plasma VL above 1000 copies/mL on two consecutive measurements within 3 months along with adherence support [23]. However, our study definition was based on published literature, allowing us to compare our findings to similar previous studies. We found no association with adherence; a primary factor for VF in previous studies [8]. We were able to characterize adherence only at cut-off levels of < 95% and ≥ 95%, as recommended by NACO. Therefore, it is possible that very few participants had truly low adherence (i.e., adherence levels < 80%) which resulted in the non-significant adjusted association with VF. Additionally, pill counting may not be the optimal method to measure adherence to second-line ART. The use of two non-invasive methods or plasma therapeutic drug concentration levels may be better adherence assessment measures [24]. We did not perform HIV genotyping to determine resistance to second-line drugs, on those with VF. However, in a study of 47 PLHIV from Mumbai (150 km from Pune), relative to drug resistance, decreased adherence was observed to be a more crucial factor for second-line associated VF [25]. This further highlights the need for better and more granular adherence measures among PLHIV receiving second-line ART. Our study is cross-sectional, limiting the exploration of causal relationships. Future longitudinal analyses are required to address this limitation. Lastly, our findings may be generalizable only to western India and more specifically Pune. We advocate for studies from different regions of India to corroborate our findings.

## Conclusions

We found second-line ART failure in 15% of our study population. In India, a combination of immunological surveillance and virological monitoring is crucial to identify patients at potential risk of VF on second-line ART. Improvements in adherence measurements and management of OIs for those on second-line ART are important to avoid VF and switch to third-line ART. Weight can be a useful clinical indicator to predict VF, but additional studies are needed to better understand the association between weight and second-line ART treatment response.

## Supplementary Information

**Additional file 1: Table S1. **Past and present opportunistic infections among adult PLHIV on second-line ART at BJGMC-SGH ART center, August 2014–September 2015.

## Data Availability

The datasets used and analyzed during the current study are available from the corresponding author on reasonable request.

## Abbreviations

ART: Antiretroviral therapy
PLHIV: People living with HIV
NACO: National AIDS Control Organization
WHO: World Health Organization
PI: Protease inhibitor
r: Ritonavir
ATV: Atazanavir
LPV: Lopinavir
NRTI: Nucleoside reverse transcriptase inhibitors
TDF: Tenofovir disoproxil fumarate
ABC: Abacavir
3TC: Lamivudine
INSTI: Integrase strand transfer inhibitor
DTG: Dolutegravir
AZT: Zidovudine
VL: Viral load
VF: Virologic failure
LMICs: Low-and-middle income countries
BJGMC-SGH: Byramjee Jeejeebhoy Government Medical College and Sassoon General Hospitals
OI: Opportunistic infection
IQR: Interquartile range
TB: Tuberculosis
OR: Odds ratio
CI: Confidence interval
aOR: Adjusted OR
