# Supplementary material for: Virological failure among people living with HIV receiving second-line antiretroviral therapy in Pune, India
Source: BMC Infect Dis. 2022 Dec 17;22:951. doi: 10.1186/s12879-022-07894-2 (PMC9758821; doi:10.1186/s12879-022-07894-2)
Supplement: Supplementary file 1 — Additional file 1: Table S1. Past and present opportunistic infections among adult PLHIV on second-line ART at BJGMC-SGH ART center, August 2014–September 2015. [file 12879_2022_7894_MOESM1_ESM.docx]

**Table S1**: Past and present opportunistic infections among adult PLHIV on second-line ART at BJGMC-SGH ART center, August 2014-September 2015

|  | Overall  (n=400) | Virological Failure^a^  (n=59) | No Virological Failure  (n=341) |
| --- | --- | --- | --- |
| **Past OI^b^ Type** |  |  |  |
| Tuberculosis | 135 | 23 (17%) | 112 (83%) |
| Diarrhea | 27 | 4 (15%) | 23 (85%) |
| Herpes Zoster | 43 | 2 (4.6%) | 41 (95.4%) |
| Candidiasis | 28 | 4 (14.3%) | 24 (85.7%) |
| Weight loss | 32 | 5 (15.6%) | 27 (84.4%) |
| Cryptococcal Meningitis | 5 | 0 | 5 (100%) |
| Others | 21 | 6 (28.6%) | 15 (71.4%) |
| **Number of past OI** |  |  |  |
| 1 | 162 | 24 (14.8%) | 138 (85.2%) |
| 2 | 53 | 6 (11.3%) | 47 (89.7%) |
| 3 | 9 | 3 (33.4%) | 6 (66.6%) |
| **Present OI^c^ Type** |  |  |  |
| Tuberculosis | 8 | 5 (62.5%) | 3 (37.5%) |
| Diarrhea | 2 | 2 (100%) | 0 |
| Herpes Zoster | 2 | 0 | 2 (100%) |
| Candidiasis | 3 | 2 (66.7%) | 1 (33.3%) |
| Weight loss | 2 | 1 (50%) | 1 (50%) |
| Cryptococcal Meningitis | 1 | 0 | 1 (100%) |
| Progressive Multifocal Leucoencephalopathy | 1 | 1 (100%) | 0 |
| Skin Infection | 1 | 1 (100%) | 0 |
| CMV Retinitis | 1 | 0 | 1 (100%) |
| **Number of present OI** |  |  |  |
| 1 | 19 | 9 (47.4%) | 10 (52.6%) |
| 2 | 0 | 0 | 0 |
| 3 | 1 | 1 (100%) | 0 |

Abbreviations: PLHIV, People living with HIV; ART, Antiretroviral therapy; BJGMC-SGH, Byramjee Jeejeebhoy Government Medical College and Sassoon General Hospitals; OI, Opportunistic Infection; CMV, Cytomegalovirus.

^a^ Defined as viral load >1000 copies/mL after at least 6 months on a second-line regimen.

^b^ Defined as occurrence of OI prior to initiation of second line ART.

^c^ Defined as occurrence of OI after initiation of second line ART.
